# Older adults’ acceptance of a robot for partner dance-based exercise

**DOI:** 10.1371/journal.pone.0182736

**Published:** 2017-10-18

**Authors:** Tiffany L. Chen, Tapomayukh Bhattacharjee, Jenay M. Beer, Lena H. Ting, Madeleine E. Hackney, Wendy A. Rogers, Charles C. Kemp

**Affiliations:** 1 Department of Biomedical Engineering, Georgia Institute of Technology, Atlanta, GA, United States of America; 2 Department of Computer Science and Engineering, University of South Carolina, Columbia, SC, United States of America; 3 College of Social Work, University of South Carolina, Columbia, SC, United States of America; 4 School of Psychology, Georgia Institute of Technology, Atlanta, GA, United States of America; 5 Department of Biomedical Engineering, Emory University School of Medicine, Atlanta, GA, United States of America; 6 Department of Medicine, Emory University School of Medicine, Atlanta, GA, United States of America; 7 Department of Medicine, Atlanta VA Geriatric Research Education and Clinical Center, Atlanta, GA, United States of America; 8 Department of Applied Health Sciences, University of Illinois at Urbana-Champaign, Champaign, IL, United States of America; University of Vermont, UNITED STATES

## Abstract

Partner dance has been shown to be beneficial for the health of older adults. Robots could potentially facilitate healthy aging by engaging older adults in partner dance-based exercise. However, partner dance involves physical contact between the dancers, and older adults would need to be accepting of partner dancing with a robot. Using methods from the technology acceptance literature, we conducted a study with 16 healthy older adults to investigate their acceptance of robots for partner dance-based exercise. Participants successfully led a human-scale wheeled robot with arms (i.e., a mobile manipulator) in a simple, which we refer to as the Partnered Stepping Task (PST). Participants led the robot by maintaining physical contact and applying forces to the robot’s end effectors. According to questionnaires, participants were generally accepting of the robot for partner dance-based exercise, tending to perceive it as useful, easy to use, and enjoyable. Participants tended to perceive the robot as easier to use after performing the PST with it. Through a qualitative data analysis of structured interview data, we also identified facilitators and barriers to acceptance of robots for partner dance-based exercise. Throughout the study, our robot used admittance control to successfully dance with older adults, demonstrating the feasibility of this method. Overall, our results suggest that robots could successfully engage older adults in partner dance-based exercise.

## Introduction

Robots have the potential to help older adults perform healthy activities, which could lead to improved health and greater independence. In this paper, we consider the possibility of robots engaging in partner dance with older adults as a form of preventive healthcare. Dance can confer mental and emotional benefits in addition to physical benefits [1, 2] and is recommended for older adults to increase their ranges of motion [3]. We focus on partner dance, which involves two dancers moving while in physical contact. Researchers have shown that partner dance can improve balance and gait for older adults [4–6]. Robot dance partners could potentially confer some of the benefits of human-human partner dance. Robots might also be given distinctive capabilities, such as making objective measurements related to human performance and health, allowing customization for individual’s preferences, and being available on demand for individual use. Robots could potentially complement human-human dance, giving older adults an opportunity to engage in the activity more frequently and conveniently.

While robots for partner dancing might have benefits for older adults, there has been a lack of research in this area. In this paper, we focus on the following three research questions:

- **Question 1**: Are older adults accepting of a robot for partner dance-based exercise?
- **Question 2**: What are facilitators and barriers to acceptance of a robot for partner dance-based exercise for older adults?
- **Question 3**: Is it feasible to use an admittance controller for partner dance-based exercise for older adults?

**Question 1:** The success of robots for partner dance-based exercise would strongly relate to their frequency of use by older adults. Prior research based on the technology acceptance model (TAM) has shown that perceived usefulness (PU), perceived ease of use (PEOU), and perceived enjoyment (PENJ) are predictive of technology usage [7–9]. We adapted methods from the technology acceptance literature to conduct a study with 16 healthy older adults to investigate their acceptance of robots for partner dance-based exercise. As part of our study, we made use of adapted measurement scales for PU, PEOU, PENJ, and other pertinent constructs.

Partner dance involves close physical interactions between robots and older adults, which could be a barrier to acceptance of this technology. There has been a lack of research that involves older adults making physical contact with human-scale robots. While there has been previous work developing human-scale robot dance partners that can follow or lead a human [10–14], they have not been formally evaluated with target users, such as older adults. To investigate the role of this close physical interaction, we assessed participants’ acceptance of robots for partner dance-based exercise before and after they physically interacted with a human-scale robot in a simple dance step we refer to as the Partnered Stepping Task (PST).

**Question 2:** More generally, understanding facilitators and barriers to older adults partner dancing with robots could help inform the design of robots to serve this role [15]. To identify facilitators and barriers to acceptance, we analyzed the results of structured interviews that we conducted with older adults after they had performed the PST with the robot.

**Question 3:** Little is known about how to design controllers that enable older adults to successfully dance with human-scale robots. In our previous work [16], we demonstrated that a simple admittance controller could allow nurses to guide a robot through navigation and positioning tasks. The robot had compliant arms and the robot’s mobile base moved with a velocity that was proportional to the force applied to the robot’s end effectors, which is a type of admittance control. We evaluated a similar controller with expert dancers who performed the PST with the same robot [17]. The robot in our study with older adults used a similar controller and a similar robot to perform the PST with older adults, providing more evidence for the feasibility of using admittance controllers for this type of human-robot interaction.

## Related work

In this section, we begin by discussing research related to our robot for partner dance-based exercise. We then describe relevant research from the technology acceptance literature. Next, we discuss research related to the barriers and motivators to exercise for older adults, followed by research on the physical and mental benefits of human-human partner dance. We also discuss socially assistive robots that have been used with older adults involving exercise. Then, we describe previous implementations of robots dancing with humans and how they relate to our work.

### Partner dance-based exercise with a robot

**Fig 1:**
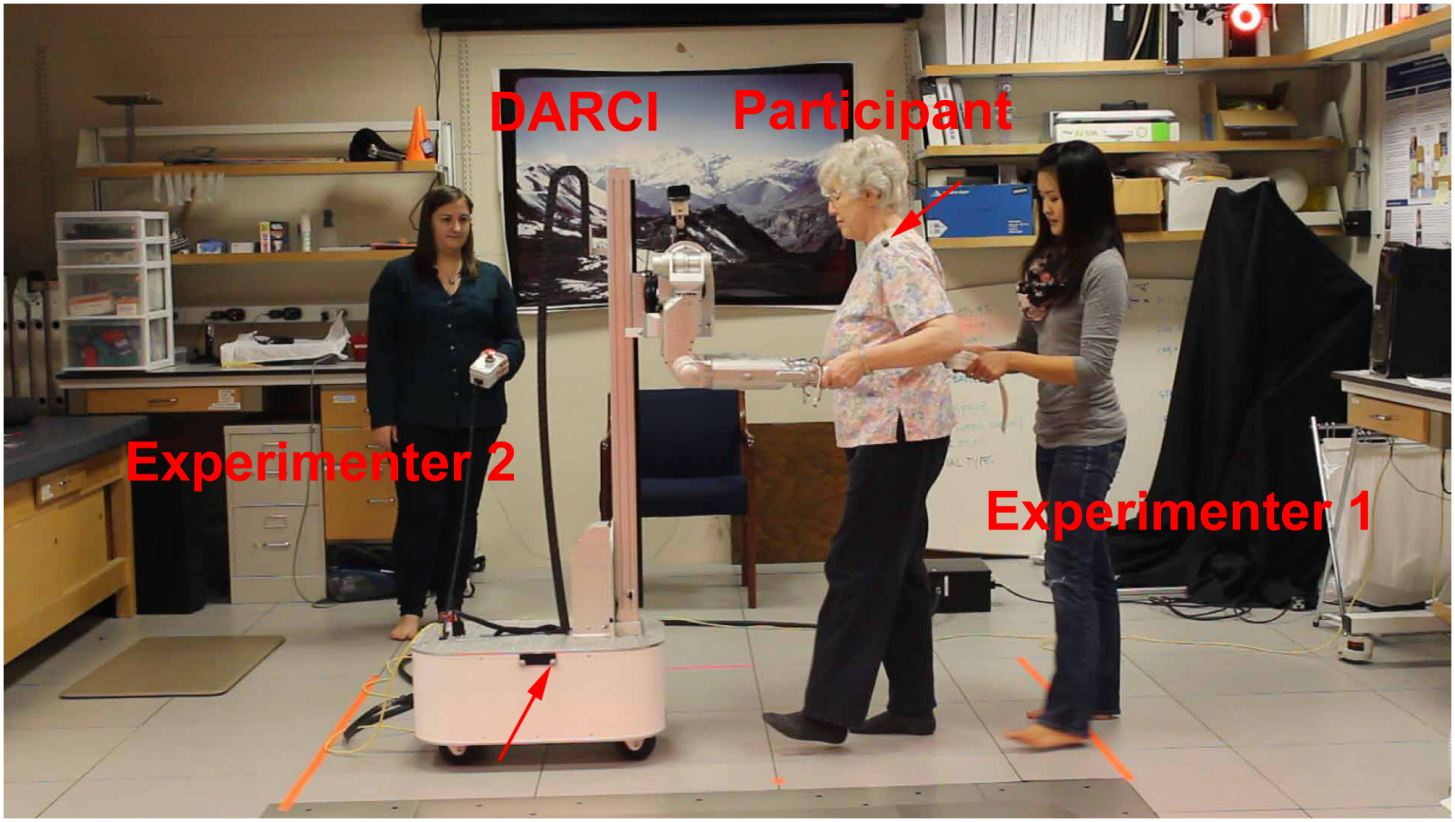
Experimental setup. Red arrows denote locations of tracking markers used in biomechanical analysis. Experimenter 1 holds gait belt placed on participant. Experimenter 2 holds run-stop button.

In our study, participants successfully led a human-scale wheeled robot with arms (i.e., a mobile manipulator) in a simple forward/backward walking dance step (see Fig 1), which we refer to as the Partnered Stepping Task (PST). Walking backwards and forwards is a fundamental component of partner dance [18]. The participants’ activities were comparable to walking at a slow speed. Light physical activities, including walking at slow speeds, have been associated with health benefits in older adults in a number of studies [19–26]. Sedentary older adults, in particular, could potentially benefit from this form of activity.

Caspersen et al. [27] define exercise as “a subset of physical activity that is planned, structured, and repetitive and has as a final or an intermediate objective the improvement or maintenance of physical fitness.” As such, the PST in our study could reasonably be used as a form of light exercise by older adults, since one could plan to perform it as a structured and repetitive activity with the objective of improving physical fitness. It might also help older adults follow walking recommendations provided by the American College of Sports Medicine (ACSM) and the American Heart Association (AHA) [28].

More generally, ballroom dancing is listed as a moderate intensity sport or recreational activity on the Physical Activity Scale for the Elderly (PASE) [29]. Robots developed for partner dance-based exercise could potentially engage older adults in more strenuous physical activities than the PST we used in our study.

### Technology acceptance model (TAM)

**Fig 2:**
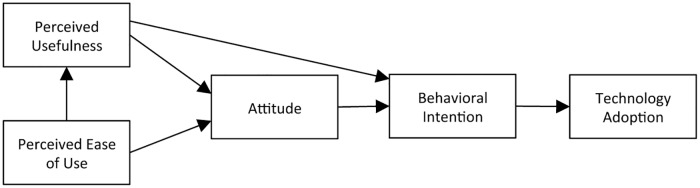
The technology acceptance model (TAM) [7].

The technology acceptance model (TAM, Fig 2) was designed to explain computer usage behavior. It has been used during the design and implementation of information technology (IT) in industry to improve use of the technology by IT employees [7]. The technology acceptance model (TAM) defines the causal linkages (correlations) between the perceived usefulness (PU) and perceived ease of use (PEOU) of a technology, the attitude (ATT) toward a technology, the behavioral intention to use (ITU) and the actual adoption (usage), of a technology [7]. More detailed definitions of these beliefs are given in Table 1. These beliefs or behaviors are also shown as boxes in Fig 2 and their empirically determined causal linkages are shown as arrows. Given these definitions and linkages, the technology acceptance model (TAM) was shown to be predictive of user behavior and to be explanatory so that researchers could identify areas of the technology that needed improvement [7]. There have been numerous extensions and permutations of the TAM model including TAM2 [30], UTAUT [31], and UTAUT2 [32] but the fundamentals remain the same—perceived ease of use and perceived usefulness (although sometimes labeled differently) are valid and reliable predictors of attitudes and intentions [15, 33, 34], and technology use [31, 32].

**Table 1:** Definitions of constructs used in the Robot Opinions Questionnaire.

Construct	Definition
Perceived Usefulness (PU)	‘The user’s subjective probability that using the technology will increase his or her performance.’ [36]
Perceived Ease of Use (PEOU)	‘The degree to which the user expects that using the technology would be free of effort.’ [36]
Attitude (ATT)	‘An individual’s positive or negative feelings (evaluative affect) about using the technology.’ [36]
Intention to Use (ITU)	‘The strength of one’s intention to perform a specific behavior to use the technology.’ [36]
Perceived Enjoyment (PENJ)	‘The extent to which the activity of using the technology is perceived to be enjoyable in its own right, apart from any performance consequences that may be anticipated.’ [8]

In this work, instead of the acceptance of IT, we were interested in the acceptance of a robotic dance partner by older adults. This could inform robot design and facilitate its adoption. In previous work, the perceived usefulness (PU) and perceived ease of use (PEOU) of robots have also been found to be informative for predicting the intention to use robots by older adults [34]. Also, usefulness and ease of use were found to predict attitudinal and intentional acceptance of a robot for both younger and older adults [35]. The technology acceptance model (TAM) has been adapted for use when determining older adults’ acceptance of a human-scale mobile manipulator to perform home-based tasks [15]. Similarly, we also adapted questionnaire items from the technology acceptance model (TAM) and other sources to understand the factors influencing older adults’ acceptance of our robotic dance partner for the specific purpose of improving and maintaining their health.

In this paper, we adapt Likert type questionnaire items from several sources [8, 15, 34, 36–38] to generate the Robot Opinions Questionnaire (Table 2). We used the Robot Opinions Questionnaire to understand the factors influencing the older adults’ acceptance of the robot both before and after interacting with it. Van der Heijden showed that when the primary purpose of software is for entertainment that the perceived enjoyment (PENJ) and perceived ease of use (PEOU) of that technology can be stronger predictors of intention to use that technology than perceived usefulness [8]. PENJ has also been found to influence older adults’ intention to use a robot [9]. Because partner dance with a robot could be construed as entertainment, we included questionnaire items regarding the perceived enjoyment (PENJ) in addition to the constructs in the original technology acceptance model (TAM).

**Table 2:** Robot Opinions Questionnaire.

PU	1.	Using a robot for partner dance-based exercise would improve and maintain my health.
	2.	I would find a robot for partner dance-based exercise useful for improving and maintaining my health.*
	3.	Using a robot for partner dance-based exercise would increase my productivity in improving and maintaining my health.
	4.	Using a robot for partner dance-based exercise would make it easier to improve and maintain my health.
	5.	Using a robot for partner dance-based exercise would enhance my effectiveness in improving and maintaining my health.
	6.	Using a robot for partner dance-based exercise would enable me to improve and maintain my health more quickly.
PEOU	7.	I would find a robot for partner dance-based exercise easy to use.*
	8.	I would find it easy to get a robot for partner dance-based exercise to do what I want it to do.
	9.	It would be easy for me to become skillful at using a robot for partner dance-based exercise.
	10.	Learning to operate a robot for partner dance-based exercise would be easy for me.
	11.	My interaction with a robot for partner dance-based exercise would be clear and understandable.
	12.	I would find a robot for partner dance-based exercise to be flexible for me to interact with.
ATT	13.	Using a robot for partner dance-based exercise would be beneficial in improving and maintaining my health.
	14.	Using a robot for partner dance-based exercise to improve and maintain my health would be a good idea.*
ITU	15.	Assuming I had access to a robot for partner dance-based exercise, I would intend to use it.*
	16.	Assuming I had access to a robot for partner dance-based exercise, I predict that I would use it.
PENJ	17.	I would find using a robot for partner dance-based exercise to be entertaining.
	18.	I would find using a robot for partner dance-based exercise to be enjoyable.*
	19.	I would find using a robot for partner dance-based exercise to be fun.
	20.	I would find using a robot for partner dance-based exercise to be pleasant.
	21.	I would find using a robot for partner dance-based exercise to be exciting.
	22.	I would find using a robot for partner dance-based exercise to be interesting.

Note: All questions measured on a 7-point scale where 1 = “Strongly Disagree,” 4 = “Neutral,” 7 = “Strongly Agree.”

*Questions discussed in detail during the structured interview.

### Older adults’ barriers and motivators to exercise

Physical exercise is commonly cited as an effective means to prevent cardiovascular disease, stroke, and diabetes [28, 39] and to improve postural and motor impairments [40] and functional performance [41] in older adults. Furthermore, the American College of Sports Medicine (ACSM) and American Heart Association (AHA) recommended that older adults perform physical activities including walking or aerobics, muscle strengthening exercises, flexibility activities, and balance exercises to maintain and improve their health [28].

However, physical activity decreases with age [42] and 87% of older adults exhibit at least one of several barriers that can prevent them from achieving the recommended level of physical exercise [43]. Schutzer and Graves identified five types of “barriers to exercise” in older adults: (1) health, (2) environment, (3) physician advice, (4) knowledge, and (5) childhood exercise. For example, poor health, a lack of exercise facilities, a lack of advice from physicians, a lack of knowledge about the value of exercise, and negative experiences with exercise in childhood can all serve as barriers [42].

Conversely, Schutzer and Graves identified four types of “motivators for exercise” in older adults: (1) self-efficacy, (2) prompts, (3) music, and (4) demographics [42]. For example, with respect to self-efficacy, Schutzer and Graves note that an older adult’s belief that he or she is capable of exercising successfully can positively influence exercise behavior. They also report that prompts through telephone calls and music during exercise have been shown to improve exercise adherence. Schutzer and Graves discuss various demographic factors associated with exercise, such as being a nonsmoker [42]. Although there is not yet a consensus for the definition of “motivation” in the rehabilitation community, rehabilitation professionals tend to agree that “motivation” is important in determining outcomes (see [44] for a literature review).

Bethancourt et al. conducted a study with 52 older adults and found that the main barriers to physical activity program participation among older adults were physical limitations due to health or aging, lack of professional guidance, and lack of awareness of suitable physical activity programs [45]. However, the motivation to maintain physical and mental health, affordability, and convenience of physical activity options served as primary facilitators [45]. Franco et al. in their review from 132 studies with 5987 older adults found six major themes: (1) social influences, (2) physical limitations, (3) competing priorities, (4) access difficulties, (5) personal benefits, and (6) motivation and beliefs, that influence participation in physical activities [46]. Schijndel-Speet et al. conducted a study with 40 older adults with mild and moderate intellectual disability and found the primary facilitators to be enjoyment, support, social contact, reward, familiarity, and routine. However, health, lack of self-confidence, lack of skills, lack of support, transportation problems, costs, and lack of physical activity options were the main barriers to physical activity participation [47]. Finally, Biedenweg et al. conducted semi-structured interviews with 39 older adults and identified marketing materials, trusted source, affordability, and location to be the frequent motivators. However, the the most common barriers were already getting enough exercise, lack of motivation, and poor health [48].

Robotics has the potential to reduce several of these barriers to exercise while enhancing motivators. In addition to playing video games with technologies such as Wii [49, 50] and Microsoft Kinect [51, 52] for increasing physical activities of older adults, video game displays or social cues have also been used to keep patients motivated during robot assisted rehabilitation or exercise [53, 54]. Furthermore, music is a fundamental element of partner dance that could serve to enhance the mood and motivate older adults. In the future, a robot could be available in the home, a senior center, or residential care facility, providing access at all times to a form of exercise.

### Benefits of human-human partner dance

Partner dance between humans has been shown to be an effective form of exercise to improve physical function in older adults. For example, McKinley et al. showed that older adults at risk for falls achieved greater improvements in balance and gait when undergoing tango dance therapy compared with therapy involving only walking [6].

Similarly, Hackney et al. showed that people with Parkinson’s disease as well as older adults who completed tango dance therapy also experienced gains in measures of balance and gait compared with traditional strength/flexibility exercise [5] or no intervention [55]. Gomes da Silva Borges et al. found that older adults living in long-term care facilities improved their functional autonomy and balance when undergoing a ballroom dancing program compared with no intervention [4].

Aside from the physical benefits of partner dance for older adults, researchers have also discussed the emotional and motivational aspects of partner dance and dance, in general. Hackney and Earhart provide a brief review of the affective and behavioral benefits of participating in dance [56]. Specifically, they mentioned that the expression of emotions through movement involved in dance can improve mood which can, in turn, improve health [57]. Among older adults with dementia living in a care facility, researchers found that dance lifted spirits, reduced agitation, and increased bonding [58]. Hackney and Earhart also mentioned that the interpersonal touch, connection, and community involvement associated with partner dance may serve as an entertaining diversion for those with physical and cognitive impairments. They also highlighted the importance of adherence to a dance program in order for older adults to receive its full benefits as with most exercise or rehabilitation programs. They report that in their previous work, participants responded favorably to tango dance therapy and were interested in continuing as evidenced in their low attrition rate [5, 55, 59, 60]. Other work has shown that an exercise program involving Korean dance movements was effective at increasing the functional status of older adults as well as motivating them to perform behaviors beneficial to their health [61].

While it is unknown whether the social and motivational benefits of dance will be seen in partner dance between humans and robots, this is an interesting area of investigation. Developing a robotic dance partner to provide partner dance therapy for older adults has the potential to confer physical and emotional benefits seen in previous human-human partner dance research. Researchers have identified various design goals for robots to effectively perform physical human-robot interactions in cooperative tasks [62, 63]. However, a critical unaddressed issue in both the partner dance therapy literature and robotic dance partner literature is whether older adults would be accepting of robotic partner dance exercise. Furthermore, the literature lacks guidance for the design of robots for this type of human-robot interaction.

### Socially assistive robotic exercise coaches for older adults

Ofli et al. designed an interactive exercise coaching system using the Microsoft Kinect and evaluated their system with six older adults. Their system showed instructional videos, monitored movements with online feedback, and recorded performance [64]. Görer et al. present a robotic fitness coach that assists older adults by performing learned gestures and using verbal instructions. It monitors the movements of older adults and adapts its instructions accordingly [65]. Gadde et al. [66] performed a study with an interactive personal trainer robot to monitor and motivate exercise in older adults. They used the robot to demonstrate exercises to participants and used a vision system to monitor their exercise activities. Fasola and Mataric provide a literature review of social robots that have been designed to assist older adults with providing information about exercise, discussing a user’s activity levels, or demonstrating exercises [54]. Specifically, previous work by Fasola and Mataric showed that a socially assistive robot (SAR) named Bandit was able to motivate and engage older adults in seated arm exercises [54]. Bandit demonstrated arm exercises and asked older adults to mimic its arm gestures. In turn, participants demonstrated arm exercises for the robot to mimic. Bandit also used facial expressions and verbal dialog to communicate. The participants completed four 20 minute sessions of exercise with Bandit over a two week period. The results of the study with Bandit provide support that robots can be used to engage older adults in exercises which could potentially be extended to other forms of social human-robot exercises such as partner dance.

Various differences between work with Bandit and our study, make our research complementary [54]. Bandit did not make physical contact with participants when demonstrating the arm exercises. In contrast, our study focuses on physical contact between the human and robot, which is a fundamental component of partner dance. Second, our work involves whole-body motion coordination during walking, which is important to partnered stepping. Third, Bandit’s height is considerably shorter than the height of the robot used in our work (seated vs. standing height). These differences in the morphology, the task, and the mode of interaction may affect participants’ responses. Finally, the primary focus of our work is to formally investigate older adults’ acceptance of robotic partner dance. While the work with Fasola and Mataric found that participants rated their interactions with Bandit to be both enjoyable and useful, we consider additional constructs from the technology acceptance literature, such as perceived ease of use and intention to use the technology (described in detail in Section 1). The hypotheses of the work with Bandit focus on the performance of the system in comparison with a computer simulation representation of Bandit on a flat-panel display as opposed to acceptance of the robot.

### Dancing robots

There have been many previous implementations of robots that dance either alone or with a human using visual interaction [67–75]. There has also been research on therapeutic robots that perform movements with or dance with older adults [54] and children [76]. However, these interactions did not involve substantial physical contact with a human partner. In our study, participants led a heavy, human-scale wheeled robot with arms in a simple forward/backward walking dance step. They did this by maintaining physical contact with the robot and applying forces to it. This is a different form of interaction with distinct implications.

Researchers have also developed partner dancing robots that make physical contact with humans [10, 14, 77–79]. However, older adults’ acceptance of these robots have not been studied. Furthermore, none of these previous robotic implementations were used in the context of exercise or health improvement. Instead, the prior work primarily focused on the ability of the robotic dance partner to follow or lead a human according to performance goals such as minimum force at the hands or minimum trajectory error.

In our previous work [17], expert dancers evaluated a robot using a simple admittance controller and generally found it to be a good follower in the context of partner dance. The robot had compliant arms and the robot’s mobile base moved with a velocity that was proportional to the force applied to the robot’s end effectors. We did not evaluate the system with older adults. For our study with older adults, we used an implementation similar to that used in [17] on a different robotic platform. We asked older adults to perform a Partnered Stepping Task (PST), a simple, forward/backward walking task, which we also used in our previous study with expert dancers.

Key distinctions from prior work are that we: (1) Asked older adults to engage in a partnered stepping interaction with a robot, and (2) Assessed the acceptance of a robotic dance partner by older adults in terms of improving and maintaining their health. We believe that these two distinctions help advance work in the area of designing therapeutic robotic dance partners for older adults.

## Implementation

In this section, we describe the robot and controller we used for this paper.

### Robot

The robot DARCI (Dynamically Adapting Robot for Cooperative Interactions) is an M1 mobile manipulator from Meka robotics. It was designed to be a general-purpose mobile manipulator. DARCI has two 7 degree-of-freedom (DoF) anthropomorphic arms, an omnidirectional base, and a 1 degree-of-freedom (DoF) vertical linear actuator to allow the robot’s torso to slide up and down. The arms have series elastic actuators (SEAs) at each of the joints, which enable low-stiffness actuation. To sense the forces at the robot’s end effectors, two 6-axis force/torque sensors are mounted at each of the robot’s wrists. Each end effector is composed of a plastic cylindrical base with a spherical rubber ball placed at the distal end to provide a handle for the participants to hold. We used similar end effectors in [16, 17]. The robot is statically stable and weighs ∼160kg.

### Controller design

We control the movement of the robot’s base using an admittance controller similar to those used in [16, 17]. Mechanical admittance is a ratio of velocity to force. An admittance controller commands velocity or position based on force [80]. For our system, the admittance controller commands the velocity of the robot’s mobile base to be proportional to the force measured at the robot’s end-effectors. The human participant applies forces to the robot’s end effectors. The robot measures and sums the forward/backward components of these forces to yield *f*_*tot*_. The controller then multiplies *f*_*tot*_ by the gain constant *c* to generate the forward/backward velocity $\dot{x}$ for the robot’s mobile base (see Eq 1).

$$\begin{array}{ccc} \dot{x} & = & c\cdot f_{tot} \end{array}$$ (1)

We set *c* to be *c* = 0.04 m/(Ns), which is larger than the higher gain setting *c* = 0.02 m/(Ns) used in [17]. We set the maximum speed to be 0.6 m/s. We also averaged the 10 most recent commands for $\dot{x}$ to reduce noise and smooth velocity transitions. When measured during development of the code, the main loop that generated these commands ran at around 100 Hz.

### Arm stiffness

In our previous work, we found that higher arm stiffness of a robot dance partner resulted in more favorable performance ratings from expert dancers [17]. Thus, we set the joints on both arms to the maximum allowed stiffness. To measure the stiffness at the end effector, we used similar methods described in [17]. The stiffness of the robot’s right arm was 465 N/m (*R*^2^ = 0.94 for the measure of goodness of fit of a linear model to the actual force and position data). This stiffness is less than the low stiffness condition that we used for the robot Cody in our previous work with expert dancers as discussed in [17]. These differences in stiffness are due to the different versions of robot arms used by the robots Cody and DARCI.

## Methodology

### Recruitment

We obtained written informed consent from all participants according to our experimental protocol that was approved by the Institutional Review Boards of the Georgia Institute of Technology and Emory University. The individual in this manuscript has given written informed consent (as outlined in PLOS consent form) to publish these case details. We recruited 16 older adults (N = 16) using the Human Factors and Aging Laboratory Participant Database at the Georgia Institute of Technology and via word of mouth. The participants were required to meet the following inclusion/exclusion criteria:

(1) US Citizen or Permanent resident (to control for the effect of culture), (2) fluent in written and spoken English, (3) 65–80 years of age, (4) able to walk without an assistive device, (5) able to use a pen to fill out questionnaires, (6) no history of falls within the last year, (7) no neurological disorders or injury, (8) no balance, vestibular, or dizziness problems, (9) no peripheral nerve injury, (10) no chronic lower back pain, numbness and/or tingling of the legs, feet, or buttock area, (11) no back or hip surgery and/or fractures within the past year, (12) no untreated anxiety disorders, and (13) no uncorrectable hearing or visual impairments.

When recruiting participants, we took care not to mention that they would be interacting with a robot, and, instead, stated they would be “interacting with technology.” We took this precaution so as to avoid recruiting participants who were biased against or in favor of interacting with a robot. Table 3 shows the participant demographics.

**Table 3:** Demographic information of participants.

**Gender**	9 female (56%), 7 male (44%)
**Age**	65–79 years, *M* = 71.5, *SD* = 5.0 years
**Ethnicity**	13 white (81%), 3 black (19%)
**Education past high school**	3 some college/Associates (19%), 5 BA/BS (31%), 6 Masters (38%), 2 Doctoral (13%)
**Marital status**	6 married (38%), 5 divorced (31%), 4 single (25%), 1 widowed (6%)
**Type of housing**	13 house/apartment/condo (81%), 3 senior housing (independent living) (19%)
**Type of transportation**	14 drive own vehicle (88%), 1 public transportation (6%), 1 no response (6%)

We administered the Mini-Mental State Examination (MMSE) and excluded participants who had an Mini-Mental State Examination (MMSE) score of less than 24, which could indicate mild cognitive impairment [81]. We did this to ensure that the participants would be able to understand the instructions for the task and the questions we asked. We excluded two participants because they achieved Mini-Mental State Examination (MMSE) scores of 21 and 22. We also excluded the data from one participant due to a robot arm failure during the initial experimental setup prior to the participant seeing or interacting with the robot. We compensated these three participants prorated for the time they participated. We did not include these three participants in the total count of N = 16.

### Procedure

We performed the experiment in the Georgia Tech Neuromechanics Lab in a climate-controlled, windowless room (see Fig 1) from December 21st, 2013 to January 21st, 2014. We asked participants to complete questionnaires in an office located near the lab as well as at a desk located inside of the experiment room.

The first author (main experimenter) read a script when conducting the experiment to maintain consistency between participants. She was also responsible for spotting participants with the gait belt by walking behind them during each trial for safety. We provide details on how the experimenter spotted the participants later in this document. Another experimenter assisted the main experimenter with checking the filled out questionnaires for completeness, running code, collecting video data, operating the run-stop button, and managing the robot’s power and data cables during the trial. For the remainder of the document we will use “we” to refer to the experimenters for convenience and readability, except where otherwise noted.

When a participant arrived, we greeted the participant, introduced ourselves, and guided the participant to a conference room. We offered the participant a snack and bottle of water. The participant read and signed a consent form and a personal health information form. Each participant also filled out reimbursement forms, demographic, and health questionnaires (adapted from [82]), and a dance experience questionnaire. They also filled out questionnaires regarding their balance confidence (Activities-specific Balance Confidence (ABC scale, [83]) and their technology experience (modified from [84]). Then, we administered the MMSE, a questionnaire to measure the older adults’ self-reported physical activity (Physical Activity Scale for the Elderly (PASE) [29]), and a questionnaire to determine the participants’ familiarity with robots (adapted from [15]). See Tables 4, 5 and 6 for the results.

**Table 4:** Participants’ previous dance experience.

**Years of general dance experience**	0.5–55 years, *M* = 13.8, *SD* = 19.6 years
**Types of general dance experience**	Ballroom, jazz, salsa, swing, line dance, ballet, tap, slow two-step, modern, fox trot
**Partner dance frequency**	4 never (25%), 6 rarely (38%), 5 occasionally (32%), 1 moderate (6%)
**Partner dance enjoyment**^†^	*M* = 4.9, *SD* = 1.9

^†^ Measured on a 7-point scale where 1 = “Strongly Disagree,” 4 = “Neutral,” 7 = “Strongly Agree.”

Note: Years and Types of general dance experience are from N = 10 participants who reported having any dance experience.

**Table 5:** Participants’ health information.

**Self-reported health rating**^‡^	*M* = 3.9, *SD* = 0.8
**Self-reported health in comparison to others**^‡^	*M* = 4.1, *SD* = 0.7
**Health satisfaction**^§^	*M* = 4.3, *SD* = 0.6
**Self-reported need to exercise more**^†^	*M* = 5.4, *SD* = 1.6
**Number of prescription medications taken**	*M* = 2.0, *SD* = 1.9
**Mini-Mental State Examination (MMSE)**	26–29, *M* = 27.9, *SD* = 1.1
**Physical Activity Scale for the Elderly** **(PASE)**^‖^	63.2–208.7, *M* = 115.4, *SD* = 40.7
**Activities-specific Balance Confidence scale** **(ABC)**^§§^	80.7–94.9%, *M* = 89.7%, *SD* = 4.0%
**Reported health conditions**	6 Arthritis, 6 Hypertension, 4 Diabetes, 1 Heart Disease 1 Other

^‡^ Measured on a 5-point scale where 1 = “Poor,” 3 = “Good,” 5 = “Excellent.”

^§^Measured on a 5-point scale where 1 = “Not at all satisfied,” 3 = “Neither satisfied nor dissatisfied,” 5 = “Extremely Satisfied.”

^†^Measured on a 7-point scale where 1 = “Strongly Disagree,” 4 = “Neutral,” 7 = “Strongly Agree.”

^‖^Score can range from 0 to 400 or more.

^§§^Measured on a 0–100% scale where 0% = “No Confidence,” 100% = “Completely Confident.”

**Table 6:** Participants’ technology experience and robot familiarity.

**Robot Familiarity**^††^	*M* = 0.25, *SD* = 0.58
**Technology Experience**^‡‡^	*M* = 12.9, *SD* = 3.8

^††^ Number of robots previously used out of a possible 13 robots.

^‡‡^Number of technologies previously used out of a possible 18 technologies.

We then led the participant to the room in the Neuromechanics Lab. We introduced the robot as a “mobile manipulator” and explained the basic function of its mobile base, vertical lift, compliant arms, and end effectors. We stated that “This robot is designed to help people who may need assistance” and that the participant should “think of how [he or she] could benefit from the use of this robot in [his or her] home or in a senior center where [he or she] might have access to it.” We instructed the participant to think of how he or she could benefit from the robot either now or in the future.

We then gave the participant an opportunity to walk around the robot and look at it from all sides. After that, we led the participant to a desk located in the room where he or she completed the Robot Opinions Questionnaire, prior to interacting with the robot (see Table 2). We will refer to this instance of the Robot Opinions Questionnaire as the *Pre* version. We then led the participant back toward the robot and explained that although the robot was capable of performing many tasks, the participant would only perform one of those tasks called “partnered stepping” with the robot. We described the definition of partnered stepping (see definition of the Partnered Stepping Task below). We explained that people can use partner dance, such as tango, waltz, salsa, or foxtrot, as a form of exercise or for entertainment purposes, or both. We mentioned that the Partnered Stepping Task (PST) was a simplified version of partner dance and was intended to give the participant an idea of what the more complex partner dance would be like. We also noted that the robot was capable of moving side-to-side and rotating, but that the study would focus on forward/backward walking.

### Partnered Stepping Task (PST)

We defined the Partnered Stepping Task (PST) in our previous work [17]. It is a simple task representative of basic coordinated motions involved in partner dance. For this study, participants performed the following specific example of a PST, which the main experimenter described and demonstrated:

- Hold onto the robot’s end effectors.
- Lead the robot backward 3 steps, starting on the right foot.
- Collect the feet together by skimming the left heel above the floor and without shifting weight onto the left foot.
- Lead the robot forward 3 steps, starting on the left foot.
- Collect the feet together. (end of one cycle)
- Repeat until four cycles are completed.
- Hold pose at the end of the last cycle until the experimenter says that it is OK to let go of the robot.

The tempo for the task was 42 beats per minute. During the task, participants listened to a synthesized drum beat at 84 beats per minute. This matches the tempo and audio in our previous study with expert dancers [17]. We noted that she would tell the participant when to start and stop so as to allow the participant to focus on the interaction between himself or herself and the robot. We told the participant that the steps need not be performed exactly right, although preferred, and that he or she could take whatever step size was most comfortable. We then guided the participant through three practice trials to learn the steps without interacting with the robot. One participant asked to perform a fourth practice trial to be comfortable with the steps.

We then placed a gait belt around the waist of the older adult. A gait belt is a device widely used in nursing. A nurse holds onto the belt to prevent a patient from falling while walking or to provide a grasping point for patient transfer [85]. Likewise, in this experiment, the gait belt provided something to grab onto to prevent the participant from falling in the event that he or she lost his or her balance. The main experimenter, who was responsible for the gait belt, held the slack of the gait belt in her left hand and held her right hand underneath the gait belt at the center of the participant’s back. She visually followed or “spotted” the participants by walking backward and forward according to the participants’ self-selected gaits.

To track the participant’s motion, we placed a reflective marker on the participant’s left shoulder and tracked it using a VICON motion capture system. We then adjusted the robot’s height using the vertical actuator at its back until the participant felt that it was comfortable. The height remained constant for all of the trials. We asked the participant to hold onto the robot’s end effectors in a symmetrical “practice frame” (see Fig 1) for increased stability and ease of use, similar to the frame used in [56]. The participant then completed one practice trial while interacting with the robot. Two participants requested to perform one additional practice trial with the robot.

We told the participant that he or she would complete three trials with the same settings as the practice trial(s) with the robot. After each trial, we administered the NASA TLX questionnaire [86] to measure workload and another task-specific questionnaire. After the participant completed all three trials, we administered the Partnered Stepping Questionnaire (Table 7). Then we led the participant back to the conference room and administered a *Post* task copy of the Robot Opinions Questionnaire as well as a Final Questionnaire. We do not analyze the task-specific questionnaire and the Final Questionnaire in this paper, since they do not address the three questions upon which we have focused.

**Table 7:** Partnered Stepping Questionnaire.

1.	The robot was a good follower.*
2.	The robot was fun to interact with.
3.	I was dancing with the robot.*
4.	I felt that the robot and I were a team.
5.	I felt a social connection with the robot.*

Note: All questions measured on a 7-point scale where 1 = “Strongly Disagree,” 4 = “Neutral,” 7 = “Strongly Agree.”

*Questions discussed in detail during the structured interview.

We then performed a structured interview based on the participants’ questionnaire responses (see end of Section 1 for details). We recorded the participants’ verbal responses using an audio recording device. We then gave the participant a copy of the experiment debriefing, consent form, and personal health information authorization form. We then thanked the participant and escorted him/her out. The entire experiment took approximately 2.5 hours.

### Subjective measures

In this section, we describe the questionnaires we used throughout the experiment to quantify the participants’ subjective experience with the robot.

As stated previously, we administered the Partnered Stepping Questionnaire (shown in Table 7) after completing all three trials with the robot. We used this questionnaire so that the participants could assess the robot’s overall performance. The questions were measured using Likert items on a 7-point scale.

We administered the Robot Opinions Questionnaire (shown in Table 2) after seeing the robot but before interacting with the robot (*Pre*) and then again after interacting with the robot (*Post*). First, we randomized the ordering of the questions. Then, we arranged the questions so that the attitude and intention to use questions were asked first in order to capture their initial reaction to the robot and to avoid being biased by the other questions. Then we shifted questions down the list to ensure that no consecutive questions were from the same construct.

To investigate the reasoning participants used to respond to the questionnaire items, we conducted a structured interview at the end of the session. During the interview, we referred back to the participants’ responses to the questions in the Robot Opinions Questionnaire *Post* and the Partnered Stepping Questionnaire denoted with * in Tables 2 and 7. For example, we stated: “For the question (state question), you responded (state participant’s rating). Please tell me more about your response.”

### Qualitative data analysis

We conducted a qualitative data analysis to systematically categorize the participants’ responses to the structured interview [87]. First, the main experimenter developed an initial “coding scheme,” or list of categories using a top-down/bottom-up approach. The top-down approach involved referring to previous literature on robot acceptance and exercise in older adults, and incorporating relevant categories according to those topics into the coding scheme (e.g., task, robot, human, environmental characteristics and exercise motivation). Then, using the bottom-up approach, the main experimenter included more specific categories that fell underneath the top-down categories (e.g., “Robot motivates/would motivate user to exercise”). Each of these specific categories is called a “code” and the process of assigning the interview responses to these categories is called “coding”. We performed this process for both potential facilitators (i.e., aspects that would encourage technology adoption) and barriers (i.e., aspects that would discourage technology adoption).

**Fig 3:**
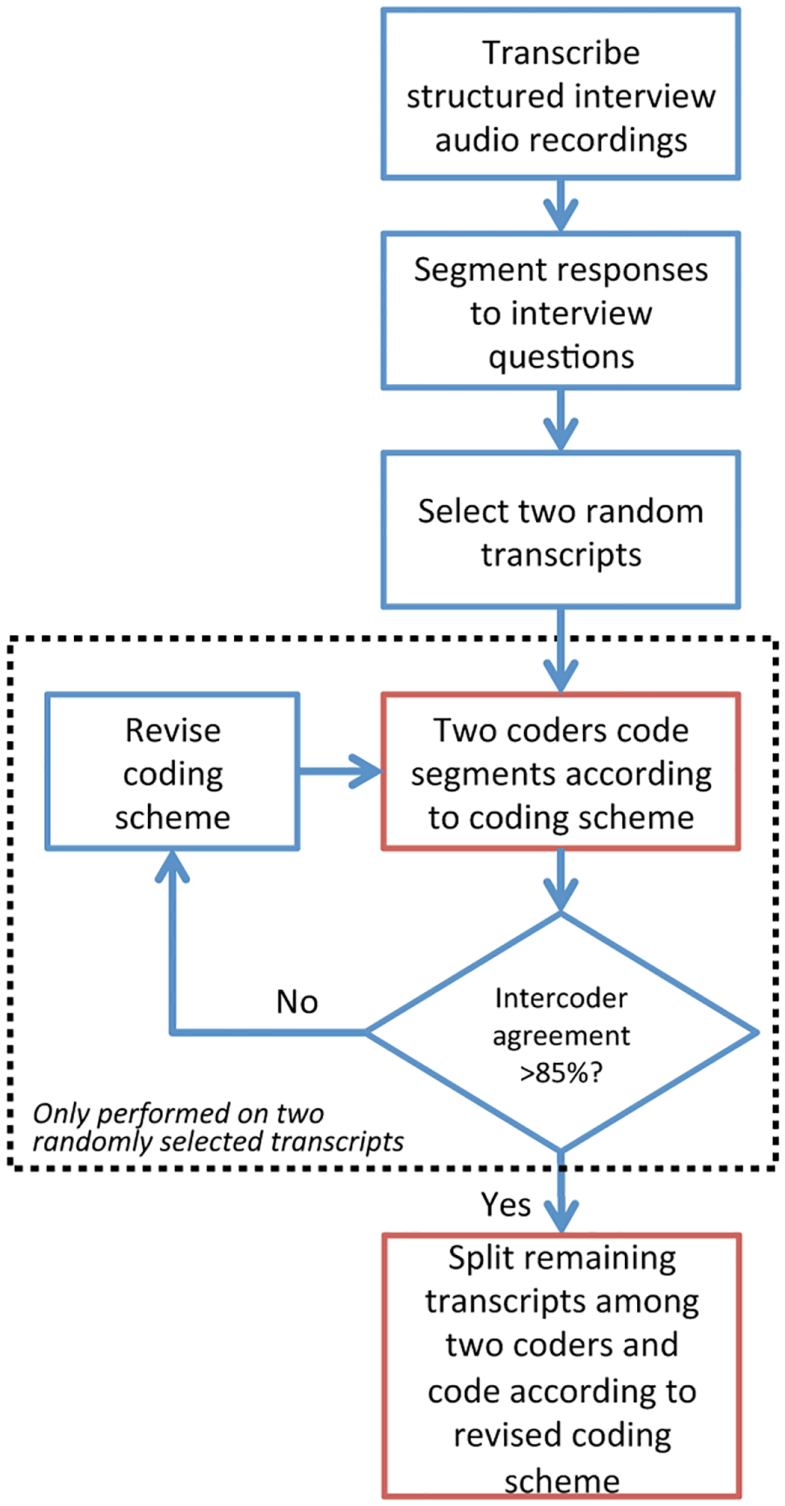
Coding process for qualitative data analysis.

We provide a diagram of the procedure we used to process the interview data in Fig 3. We transcribed the participants’ responses to the structured interview questions verbatim from audio recordings of the interviews. We loaded the transcripts into MAXQDA 11 which is a software tool used to analyze qualitative data [88]. We parsed the transcripts into “segments” where a segment was defined as a participant’s response to an interview question. Then, we randomly selected two transcripts and “coded” (categorized) the segments according to the initial coding scheme. A primary and secondary coder (the main experimenter and a fellow lab member) coded the segments of these same two transcripts. During the coding process, a coder categorized a segment as containing any number of facilitators or barriers according to the coding scheme. The primary and secondary coders completed two rounds of coding the same two randomly selected transcripts. After each round, the coders resolved discrepancies by adding, removing, and refining codes to the scheme. The third round of coding resulted in 88% intercoder agreement. 85% intercoder agreement is an acceptable minimum in qualitative research [87]. After agreement was reached, the coding scheme was finalized and not changed any further. We divided the remaining 14 transcripts evenly among the primary and secondary coders to code individually according to the final coding scheme.

## Results

In this section, we begin by describing the background information for the participants. Then, we discuss the results with respect to our three research questions in Section 1.

### Participant background information

81% of the participants identified themselves as white and 19% identified themselves as black. They reported varied levels of education and income (Table 3). 81% responded that they lived in their own homes and 19% responded that they lived in independent senior housing (19%).

10 participants reported having dance experience, which was variable, ranging from 0 years to 55 years (Table 4). When we asked all the participants if they enjoyed partner dance, their average response was 4.9 where 4 = “Neutral” and 5 = “Slightly Agree.” Participants had low experience with robots and moderate technology experience (Table 6).

Participants reported themselves to be in good health (Table 5). Their physical activity levels (Physical Activity Scale for the Elderly—PASE) were in line with reported Physical Activity Scale for the Elderly (PASE) norms [89] when separated by gender (males: *M* = 134.8, *SD* = 54.1 and females: *M* = 100.3, *SD* = 18.0). They had an average of 89.7% confidence in their balance in doing the activities listed in the ABC questionnaire where higher than 80% is associated with highly functioning, physically active older adults [90].

### Research Question 1: Older adults are accepting of a robot for partner dance-based exercise

By analyzing the participants’ responses to the *Post* Robot Opinions Questionnaires using methods from the technology acceptance literature, we found that older adults in our study were accepting of a robot for partner dance-based exercise. In this section, we discuss the statistical analysis for Research Question 1 in detail. We used non-parametric statistical inference tests as recommended in [91] throughout this paper.

We computed a Cronbach’s *α* value to measure the internal consistency of the responses to each of the constructs in the Robot Opinions Questionnaire (perceived usefulness—PU, perceived ease of use—PEOU, attitude—ATT, intention to use—ITU, perceived enjoyment—PENJ, for both *Pre* and *Post* tests for a total of 10 Cronbach’s *α* values). The Cronbach’s *α* values were between .86 and .99, indicating excellent internal consistency for each of the constructs. These results allowed us to average across the Likert ratings for each of the constructs for each participant. Table 8 reports the medians and ranges for these averages.

**Table 8:** *Pre* and *Post* acceptance results.

	**Pre**				
**Construct**	**Mdn**	**Range**	*Z*	*r*	*p*
PU	5.8	3–7	3.05	.76	.002**
**PEOU**	**4.3**	**2.8–6**	**2.63**	**.66**	.009**
ATT	6	4–6.5	3.37	.84	<.001***
ITU	6	3–7	3.30	.82	<.001***
PENJ	5.3	3.7–6.3	3.05	.76	.002**
	**Post**				
**Construct**	**Mdn**	**Range**	*Z*	*r*	*p*
PU	6	1.2–7	2.51	.63	.012*
**PEOU**	**6**	**3.7–7**	**3.42**	**.85**	<.001***
ATT	6	1.5–7	2.46	.62	.014*
ITU	6	1.5–7	2.38	.59	.017*
PENJ	5.8	1.7–7	2.61	.65	.009**

Note: All tests are Wilcoxon signed-rank tests with a test score of 4 = “Neutral.” Refer to Table 2 for complete questions.

*p<.05,

**p<.01,

***p<.001

The purpose of the following analyses was to determine whether the participants’ acceptance ratings of the robot were significantly different than “Neutral.” Table 8 shows the results of the Wilcoxon signed-rank tests (test score of 4 = “Neutral”) for the responses to the *Pre* and *Post* Robot Opinions Questionnaire. The data show that the participants had acceptance ratings that were significantly above a neutral response (*α* = .05), across all 5 constructs of acceptance, for both the *Pre* and *Post* acceptance measurements.

**Fig 4:**
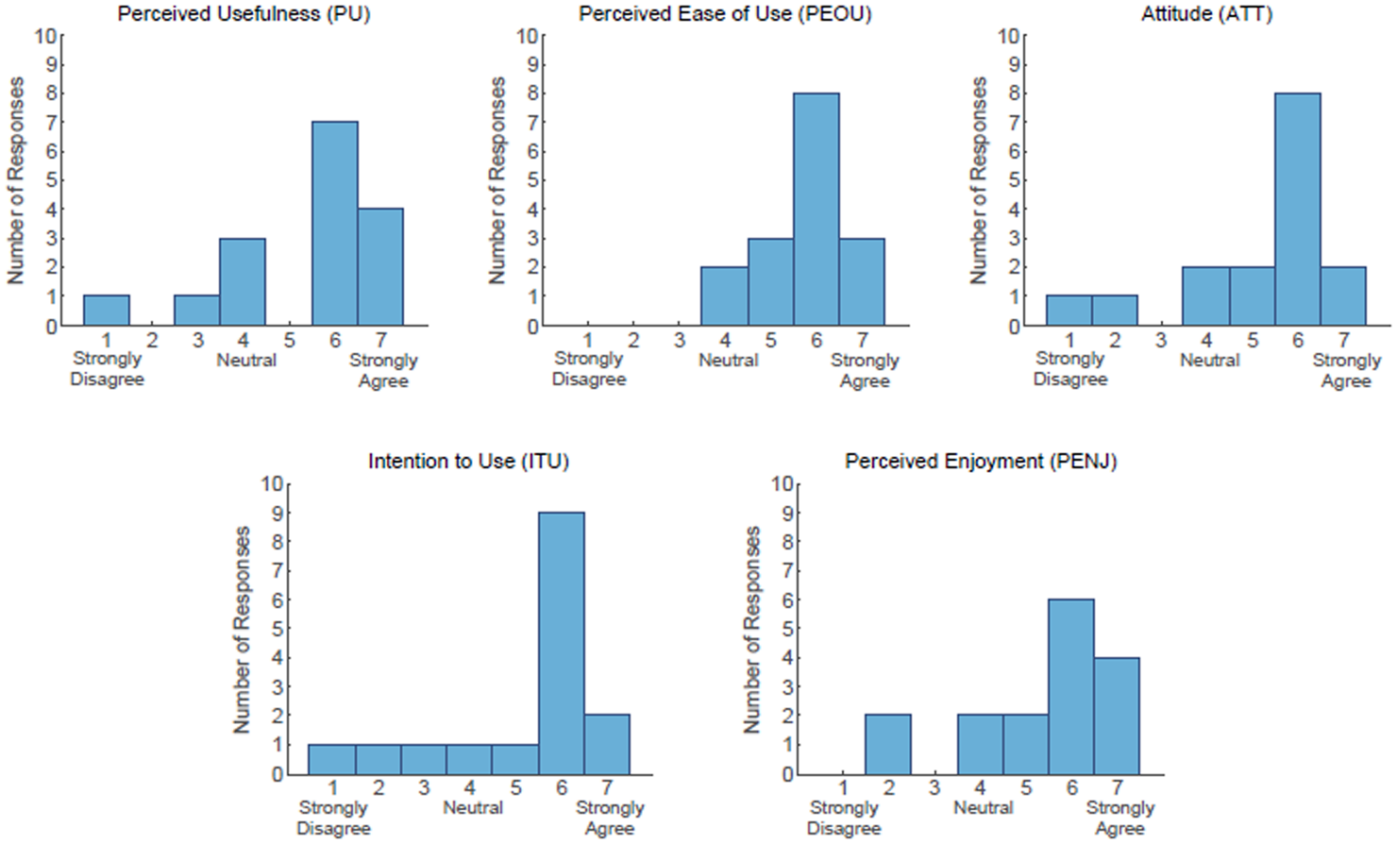
Histograms of responses to Robot Opinions Questionnaire (*Post*, overall scale) asked during interview.

These results indicate that the participants were accepting of the robot for parter dance-based exercise both before as well as after physically interacting with the robot with the PST. For the *Post* responses to the Robot Opinions Questionnaire, the median responses to each of the 5 constructs of acceptance were either 5.8 or 6 where 5 = “Slightly Agree,” and 6 = “Agree” on the 7-point scale. Fig 4 shows histograms of the *Post* responses to the Robot Opinions Questionnaire.

Notably, participants’ perceived ease of use (PEOU) significantly increased after performing the PST with the robot, changing from the *Pre* (Mdn = 4.3) to the *Post* (Mdn = 6). As shown in Fig 4, after the PST no PEOU construct rating was lower than a 4 = “Neutral” and 14 out of the 16 participants had PEOU ratings between 5 and 7, which correspond with varying levels of agreement. In contrast, each of the other construct ratings had at least two ratings below 4 = “Neutral” after the PST, which corresponds with some level of disagreement.

Table 9 shows the results of the *Pre* vs. *Post* Wilcoxon signed-rank tests we performed to assess changes in responses after the PST. While the PEOU significantly increased, we found no significant changes (*α* = .05) for the other four Robot Opinions Questionnaire constructs.

**Table 9:** Comparing *Pre* vs. *Post* acceptance results.

Question	Pre Median	Post Median	*Z*	*r*	*p*
PU	5.8	6	0.57	.14	.57
**PEOU**	**4.3**	**6**	**3.24**	**.81**	.0012**
ATT	6	6	-0.43	.11	.66
ITU	6	6	-0.31	.08	.76
PENJ	5.3	5.8	1.43	.36	.15

Note: All tests are Wilcoxon signed-rank tests. Refer to Table 2 for complete questions.

**p<.01

### Research Question 2: Facilitators and barriers provide insight on acceptance

By performing a qualitative data analysis (described in Section 1) on the participants’ responses during the structured interview, we identified several facilitators and barriers to older adults’ acceptance of a robot for partner dance-based exercise. Of note, participants found the robot easy to use, which supports the findings for Research Question 1. Furthermore, participants generally mentioned more facilitators than barriers.

Tables 10 and 11 show facilitators and barriers, respectively, that participants mentioned during the structured interview. Specifically, the data in these tables are only from the interview responses when asking participants to elaborate on their responses to the five questions of the Robot Opinions Questionnaire *Post* denoted by a * in Table 2. The counts in Tables 10 and 11 show the number of participants who mentioned a specific facilitator or barrier at least once over these five questions. We only show facilitators or barriers that were mentioned by at least three participants.

**Table 10:** Facilitators of robot acceptance of a robot for partner dance-based exercise.

Rank	Facilitator	# of people who mentioned
1	Robot is easy to use	11
2	Robot is enjoyable	8
3	Robot motivates/would motivate user to exercise	6
3	Robot would improve health (general)	6
4	Robot performed task well (general)	5
5	Can use robot when human partner is not available	4
5	Robot provides/would provide a means to exercise	4
5	User likes to dance | User wants to learn how to dance	4
6	Robot does exactly what it is told	3
6	Robot is/would be always available	3
6	Task was simple | easy to learn	3

Note: These are facilitators mentioned by at least three people during structured interviews regarding participants’ responses to questions in Table 2 denoted with* (Robot Opinions Questionnaire *Post*).

**Table 11:** Barriers of robot acceptance of a robot for partner dance-based exercise.

Rank	Barrier	# of people who mentioned
1	Task does not provide exercise | would not improve health	5
2	Robot does not do/teach new dance moves/exercises	4
2	Robot is not enjoyable	4
2	Task was too simple | boring	4
3	User does not need/want robot (general)	3

Note: These are barriers mentioned by at least three people during structured interview regarding participants’ responses to questions in Table 2 denoted with* (Robot Opinions Questionnaire *Post*).

Out of the facilitators and barriers we identified, the robot being easy to use was mentioned by the most participants (i.e, 11 out of the 16 participants). This result reinforces our finding in Research Question 1 that the robot was perceived to be significantly easier to use after performing the PST with the robot. For example, one participant stated that the robot was “light to the touch,” and that “as I moved, the robot moved with me, with no trouble at all,” while another participant said it was “very easy to do so, to control it” and that “there was no problems [*sic*] whatsoever.” Participants also expressed that the robot performed the task well (5) and did exactly what it was told (3). For example, one participant said: “It just simply followed my instructions.” One participant even compared the robot with his girlfriend: “It never fought me, it never tried to move in the direction like my old girlfriend, wanting to go in a different direction than I wanted to go.”

While the robot was perceived as easy to use, the simplicity and lack of variation in the task came up as potential barriers. Three participants stated that the task was simple or easy to learn while 4 expressed dissatisfaction with the task’s simplicity. For example, one participated stated: “I didn’t feel as though it was difficult for me to grasp what was necessary to do. I didn’t feel confused or uncomfortable in any way.” This participant expressed concern that technology that was too complicated and would not be adopted by people older than he was. On the other hand, another participant stated “I couldn’t do that for a long period of time, it’s boring.” Along similar lines, 4 participants mentioned that the robot did not do or teach new dance moves or exercises. For example a participant said: “I would go out of my way to use [the robot], you know, if it included learning dances and new steps. I think that would be very enjoyable.”

A number of participants noted facilitators related to health and exercise. Several participants mentioned that the robot would improve their health (6) and would provide a means to exercise (4). For example, one participant stated that “if I didn’t use a robot or have self-imposed exercises, my health would decline,” and another stated that the robot “is good for the eye-hand coordination and the brain coordination with the physical body.” One participant stated: “I would use [the robot] on a daily basis, while I’m watching the news … I don’t have an exercise machine, but [the robot] would be my exercise machine, to dance … to raise the heart rate.” 6 participants mentioned that the robot would motivate them to exercise. For example, one participant stated: “because of the reliability that it would be there for me whenever I look at it, that would encourage me more, ‘hey, let’s dance!’” One participant said that the robot would “try to encourage you instead of like a piece of furniture” by engaging in spoken dialog and saying: “‘don’t be lazy!’ or ‘oh I know you’ll feel better when you’re finished.’”

However, 5 participants stated that the task performed would not provide exercise or improve health. For example, one participant said: “I don’t find that exercising at all, it was very little … compared to what I do.” This participant said that he walks 1–3 miles per day as exercise.

While 8 participants stated that the robot was enjoyable (facilitator), 4 mentioned that it was not (barrier). For example, some participants stated that “I thought it was great fun, and it would encourage me to do more dancing,” or that “it would encourage me to exercise more and it was fun…I enjoy walking more than I do lifting weights.” At the same time, another participant stated “what could be more exciting about putting dishes in the dishwasher, am I supposed to get excited about that? I consider the robot like a dishwasher.” In a related facilitator, 4 participants expressed that they either liked to dance or would want to learn how to dance. For example, one participant stated: “I love dancing so … if it were only the robot would be available to dance then we would dance [*sic*].”

On their own, several participants recognized that a robot dance partner could potentially be more available and convenient than human dance partners, and indicated that they perceived value in this potential attribute. Three participants mentioned that the robot would always be available and 4 specifically mentioned that they could use a robot when a human partner was not available. For example, one participant stated: “Consider if…you have bad weather out, and you can’t get to any place where you’re going to get exercise. The robot would be there to take up your interest.” Another mentioned that if his girlfriend was not able to accompany him to their dance class, “if I could buy a robot to teach me at home, I would do that.”

On the other hand, three participants simply expressed that they did not want or need a robot. For example, one participant stated: “if I had a stroke, then I might find someplace to do this. I have not had a stroke so I think it’s too slow and I would not participate with it.”

In summary, participants expressed a variety of facilitators and barriers when discussing their responses to the Robot Opinions Questionnaire *Post*. These facilitators and barriers could potentially help guide future designs of robots for partner dance-based exercise for older adults. Generally, more participants mentioned facilitators than barriers, which captures the generally favorable acceptance ratings associated with Research Question 1.

### Research Question 3: Older adults successfully completed partner dance-based exercise with a robot using an admittance controller

To determine the feasibility of using an admittance controller for partner dance-based exercise with older adults, we assessed whether the participants were able to complete the task with the robot as instructed. We also asked to what extent the participants rated the robot as performing the task well. We discuss several objective task measures in this section to assess performance of the participants and robot. Also, we refer to the responses to the Partnered Stepping Questionnaire (Table 7) to determine the participants’ subjective assessment of the robot’s performance. While we asked the participants to perform the task preferably in the way the experimenter instructed, we informed them that it was more important to focus on the interaction between them and the robot.

**Fig 5:**
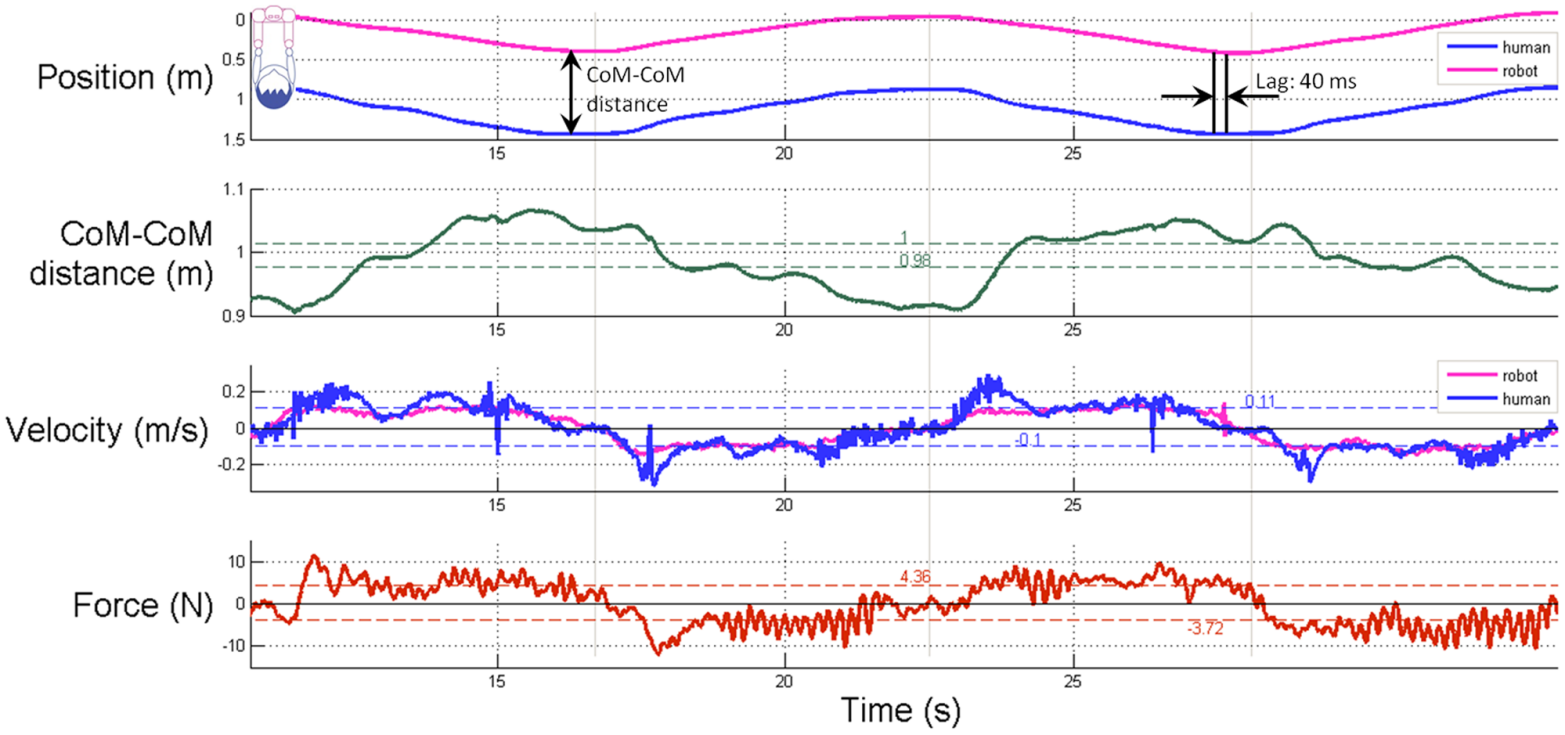
Biomechanics of human-robot partnered stepping. Example data from two cycles of one trial from one participant. We compute the lag time (lag) by cross correlating the robot’s position as a function of time and the human’s position as function of time, where position is a scalar.

In this section, we will refer to several of the biomechanical measures we computed from the force and motion capture data. Fig 5 shows an example of the force and kinematic data collected and processed for one trial for one participant. We computed the average estimated distance between the center-of-mass of the leader to center-of-mass of the follower (CoM-CoM distance), which is the distance between the markers on the robot and human denoted by red arrows in Fig 1). We did not measure the actual centers of mass. Instead, center-of-mass (CoM) refers to a point on the robot where one would expect the base of the neck to be located and the center-of-mass (CoM) for participants refers to the shoulder motion-capture marker. We also computed the standard deviation of the CoM-CoM distance (CoM-CoM standard deviation), the velocity of the human and the robot, and the force for each trial, both when the human was walking forward and when the human was walking backward.

We found that participants were able to complete the task in a manner that closely matched the instructions given by the experimenter. After the completion of the experiment, we viewed the video recordings of the trials and manually counted the number of steps the participants took as well as the number of cycles they completed during each trial. Participants performed *M* = 25.4, *SD* = 3.2 steps per trial, where 24 steps per trial corresponds with the preferred performance. They completed *M* = 4.2, *SD* = 0.5 cycles per trial where 4 cycles corresponds with the preferred performance. As a result, they performed an average of *M* = 6.1, *SD* = 0.3 steps per cycle, where 6 steps per cycle corresponds with the preferred performance. Extra cycles and steps performed by the participants were due to the experimenter allowing a participant to complete an additional complete cycle if a participant misstepped during a trial (e.g., shuffled feet or paused) or due to experimenter miscount.

Participants traveled an average distance of *M* = 0.9, *SD* = 0.2 m per cycle, which indicates that they performed the steps in a way that resulted in translating their center-of-mass (CoM) positions during overground walking as instructed (as opposed to stepping while staying in one place). In addition, all participants maintained physical contact with the robot’s end effectors throughout each of the trials. Furthermore, no participants fell during the experiment and no adverse events occurred, so the experimenters did not provide physical support to the participants with the gait belt nor did they push the run-stop button to halt the robot.

Across all trials, the participants and the robot maintained a CoM-CoM distance of *M* = 0.98, *SD* = 0.05 m when walking forward and *M* = 1.04, *SD* = 0.04 m when walking backward. The CoM-CoM standard deviation across trials was *M* = 0.05, *SD* = 0.03 m both when walking forward and when walking backward.

The average force applied to the robot’s end effectors across all trials was *M* = -4.7, *SD* = 1.0 N (forward) and *M* = 4.9, *SD* = 1.0 N (backward). Also, the average robot velocity was *M* = -0.08, *SD* = 0.02 m/s (forward) and *M* = 0.09, *SD* = 0.02 m/s (backward). Similarly, the average velocity of the participants was *M* = -0.11, *SD* = 0.02 m/s (forward) and *M* = 0.11, *SD* = 0.02 m/s (backward).

Considered as a whole, these objective task measures indicate that participants performed the task in ways that closely followed the experimenter’s instructors. Participants applied forces to the robot’s end effectors while maintaining constant contact with the robot and completing the steps of the PST. The participant and the robot moved together during overground walking at similar speeds and maintained a relatively consistent amount of distance between them.

Table 12 shows the results of the Wilcoxon signed-rank tests (test score of 4 = “Neutral”) for the responses to the Partnered Stepping Questionnaire. Participants generally agreed that the robot was a good follower, the robot was fun to interact with, they were dancing with the robot, and that they felt the robot and them were a team. The responses to these questions had a median score of 5.5 or 6 where 5 = “Slightly Agree” and 6 = “Agree.”, indicating that the participants generally felt that the robot performed the task well.

**Table 12:** Partnered Stepping Questionnaire results.

Question	Median	Range	*Z*	*r*	*p*
**1. Good follower**	**6**	**4–7**	**3.54**	**.89**	<.001***
**2. Was fun**	**6**	**2–7**	**2.65**	**.66**	.008**
3. Was dancing	5.5	2–7	2.34	.59	.02*
4. Were a team	6	2–7	2.41	.60	.02*
5. Social connection	4.5	1–7	0.35	.09	.73

Note: All tests are Wilcoxon signed-rank tests with a test score of 4 = “Neutral.” Refer to Table 7 for complete questions.

* *p*<.05,

** *p*<.01,

*** *p*<.001

In summary, regarding Research Question 3, it is feasible to use an admittance controller for partner dance-based exercise for older adults. We found that older adults were able to complete the PST with the robot, which had compliant arms and used an admittance controller to command the velocity of its mobile base. The participants also rated the robot as performing the task well.

## Limitations

It is unclear whether our findings regarding older adults’ acceptance can generalize to long-term acceptance of partner-dance based exercise robots, as there has been little previous work modeling long-term usage [92]. Our work provides evidence that older adults would be willing to try out robots for partner-dance based exercise and that dancing with a robot can result in older adults perceiving it as being easier to use.

At the beginning of the experiment, we introduced the robot and communicated its intended purpose. This may have primed participants to respond more positively in our study. We did, however, follow standard practices for assessing perceptions of usefulness in the technology acceptance literature [36], and the facts we conveyed are comparable to facts that might be conveyed upon a commercial robot being introduced to older adults in practice. Throughout the study, the main experimenter communicated in a reserved and factual manner with the participants in order to reduce potential bias. In addition, the experimenter conveyed that the partnered stepping task that the participant would be performing with the robot was just one of many tasks the robot is capable of performing. This could potentially increase the comfort a participant would feel in providing negative feedback about the particular task of partnered stepping. Notably, not all participants were positive in their responses (See Table 11), so the introduction did not preclude critical responses from the participants.

We conducted our study using a relatively small sample size of N = 16 of older adults from the Atlanta metropolitan area in the United States, so our results may not generalize to the broader older adult population or older adults from other cultures, demographics, and geographic regions. In addition, attitudes and perceptions about new technologies may not always be directly predictive of future use (e.g., [33]). However, the findings are mixed and attitudes and perceptions are predictive of use in some cases (e.g., [31, 32]) and they do provide design guidance. Our findings regarding acceptance and the facilitators and barriers of acceptance give roboticists and human-robot interaction designers an initial guide for the design of future robotic dance partners for older adults. A recent review by Peek and his colleagues ([93]) indicated that the factors that predict technology acceptance vary over time of use (pre-experience vs. post-experience). Thus, a potential direction for future efforts would be to focus on the use of a dance robot over time, and to develop predictive models for the relative importance of different factors that influence usage behaviors over time.

During the PST, the main experimenter was responsible for spotting the participant using the gait belt for safety. This could potentially influence the participants. The main experimenter was trained to use the gait belt and practiced prior to the study. During the PST, she was careful to allow enough slack in the gait belt so as not to apply significant force, which was achievable due to the slow and predictable motions of the participants during the PST. She also remained out of the participant’s field of view. When we piloted the study with two older adults, we asked them about the gait belt. Neither pilot participant stated that he/she felt the experimenter touching or pulling on him/her during the task. Throughout the actual study, none of the participants provided negative comments about the physical contact the experimenter made with the gait belt.

While the results of our study are promising, the extent to which human-robot partner dance can be similar to human-human partner dance remains an open question. As such, human-robot partner dancing may not confer the same health benefits as human-human dancing. For example, social connection can be considered an important part of human-human partner dance, but the responses from participants in our study generally did not indicate that participants felt a social connection with the robot. Human-robot dancing and human-human dancing may be more appropriately considered as distinct, but related phenomena, with human-human dancing serving as a source of inspiration for human-robot dancing.

## Conclusion

In this work, we have demonstrated that it is feasible for older adults to lead a human-scale mobile manipulator in a simplified partner dance. For our study, we focused on three research questions: Are older adults accepting of a robot for partner dance-based exercise? What are facilitators and barriers to acceptance of a robot for partner dance-based exercise for older adults? Is it feasible to use an admittance controller for partner dance-based exercise for older adults?

The 16 older adults in our study were generally accepting of robots for partner dance-based exercise, tending to perceive it as useful, easy to use, and enjoyable. Notably, participants perceived the robot as being easier to use after dancing with it. These results suggest that older adults are open to partner dancing with a robot to improve their health.

We also identified facilitators and barriers to acceptance of robots for partner dance-based exercise based on interviews with the older adults in our study. Many participants noted that the robot was easy to use, enjoyable, and performed the task well. Participants also made positive comments about the potential benefits of the robot for health and exercise. However, some participants were not positive about the robot in terms of exercise and health, finding the activity to be too easy, boring, or lacking in physical exertion. Participants suggested that a robot could actively encourage them to exercise and teach them dances. Notably, participants identified the potential availability and convenience of a robot dance partner as a positive attribute distinct from human dance partners.

Throughout our study, the robot compliantly held its arms in fixed postures and used a simple admittance controller that commanded the velocity of its wheeled base to be proportional to the force applied to its end effectors. All 16 participants successfully performed the Partnered Stepping Task (PST) with the robot using this straightforward control method. As noted previously, they also found the robot easy to use. As such, the control method used by our robot can potentially serve as a tangible example for other control engineers to build upon in the future.

With our relatively simple system, participants tended to respond positively. However, some participants expressed the desire for a more varied dance step routine for more enjoyable interaction. This is in line with the feedback obtained by Olfi et al. [64] where participants asked to have more variety in the exercise routines for longer-term use. Future robots for partner dance-based exercise could potentially be more engaging by increasing the complexity and variety of dances. For example, a robot might allow full planar motion (i.e., rotation and 2D translation), as we studied in our earlier work [16]. Enabling robots to serve as the leader when dancing with older adults also presents interesting possibilities.

Given the importance of physical activity for healthy aging and the positive results from our study, human-robot partner dance merits further attention. Continued work in this area may one day result in a robot that helps older adults improve their health and well-being.

## Supporting information

S1 Video A video showing the robot experiments with older adults for partner dance-based exercise. (MP4)
